# Depression and Anxiety in Patients with Psoriasis: A Comprehensive Analysis Combining Bibliometrics, Latent Dirichlet Allocation, and HJ-Biplot

**DOI:** 10.3390/healthcare13050441

**Published:** 2025-02-20

**Authors:** Aline Siteneski, Karime Montes-Escobar, Javier de la Hoz-M, German Josuet Lapo-Talledo, Geovanna Gutiérrez Moreno, Esther Carlin Chavez, Rosangela Caicedo Quiroz, Gulnara Patricia Borja-Cabrera

**Affiliations:** 1 Faculty of Health Sciences, Medicine Career, and Research Direction, Universidad Técnica de Manabí, Portoviejo 130105, Ecuador; 2 Departamento de Matemáticas y Estadística, Facultad de Ciencias Básicas, Universidad Técnica de Manabí, Portoviejo 130105, Ecuador; kmontes@usal.es; 3 Facultad de Ingeniería, Universidad del Magdalena, Santa Marta 470004, Colombia; jdelahoz@unimagdalena.edu.co; 4 Specialization in Occupational Health and Safety, Faculty of Medicine, Pontificia Universidad Católica del Ecuador, Portoviejo 130150, Ecuador; gerjolata@gmail.com; 5 Derma Center, Portoviejo 130102, Ecuador; geovannaderma@hotmail.com; 6 Centro del Cuidado Integral y Promoción de la Salud, Universidad Bolivariana del Ecuador, Duran 092405, Ecuador; elcarlinc@ube.edu.ec (E.C.C.); rcaicedoq@ube.edu.ec (R.C.Q.); 7 Escuela de Salud, Facultad de Posgrados, Universidad Estatal de Milagro—UNEMI, Milagro 091050, Ecuador; 8 Escuela de Medicina, Colegio de Ciencias de la salud, Universidad San Francisco de Quito, Quito 170901, Ecuador

**Keywords:** psoriasis, depression, anxiety, bibliometric analysis, Latent Dirichlet Allocation (LDA), psychiatric comorbidity, HJ-Biplot

## Abstract

**Background:** Patients with psoriasis often experience psychiatric comorbidities, such as depression and anxiety. These comorbidities can lead to poorer adherence to treatment regimens, reduced effectiveness of therapies, and a heightened disease burden. This study aims to explore the scientific output related to psoriasis, depression, and anxiety using a comprehensive analysis combining bibliometric statistical methods. **Methods:** The study performed a bibliometric analysis of publications related to psoriasis, depression, and anxiety between 1974 and December 2023. This study employed the Latent Dirichlet Allocation (LDA) algorithm to identify key research topics and used the HJ-Biplot technique to visualize the relationships between publications and research indicators. The inclusion criteria were limited to English-language research articles. **Results:** Over 49 years, the analysis identified 5059 documents published across 1151 sources. The annual growth rate for research was 12.26%. *The Journal of the European Academy of Dermatology and Venereology* and *The British Journal of Dermatology* were found to be the leading journals in this field. The United States emerged as the top contributor, followed by China, Italy, and Germany. The most prevalent research topics were inflammation and cellular function, with a significant focus on patient treatment and the impact of depression and anxiety. **Conclusions:** This bibliometric analysis underscores the increasing of studies on the comorbidities of depression and anxiety in patients with psoriasis. This study provides a comprehensive overview of research trends and emerging topics in this field, offering valuable insights for future investigations.

## 1. Introduction

Psoriasis is an immunologically mediated, remitting–relapsing, inflammatory disease that affects the skin, nails, and joints [1]. Psoriasis imposes significant physical, emotional, and social burdens that directly affect patients’ quality of life [2]. Individuals with psoriasis exhibit a 40% to 90% higher prevalence of psychosocial comorbidities compared to the general population [2]. Stress, substance abuse, anxiety, and depression are among the most prevalent psychosocial comorbidities closely linked to psoriasis [3]. Previous studies have reported that psoriasis may precede mental health diagnoses and is particularly associated with increased rates of anxiety, depression, and newly diagnosed bipolar disorder [4,5]. Moreover, disease severity may be correlated with stress, depressive disorders, and suicidal ideation [6]. The relationship between psoriasis and these psychiatric disorders is complex, bidirectional, and influenced by biological, psychological, and social factors. Biologically, psoriasis and mental health disorders share common inflammatory pathways [7]. Dysregulation of the hypothalamic–pituitary–adrenal (HPA) axis, commonly observed in chronic stress and depression, has also been implicated in the exacerbation of psoriatic lesions [8]. Recent studies suggest that the relationship between psoriasis, depression, and anxiety may also have genetic underpinnings [9,10]. The interplay of these factors underscores the importance of considering psoriasis not solely as a dermatological condition but as a systemic disease with significant mental health implications.

The proportion of patients with psoriasis presenting with clinical depression is 19% according to the DSM-IV (*Diagnostic and Statistical Manual of Mental Disorders*, Fourth Edition) and 12% according to the International Classification of Diseases (ICD) criteria [11]. The use of antidepressants in patients with psoriasis suffering from depression is low, approximately 9% [11]. It is estimated that more than 10% of patients with psoriasis have clinical depression, and around 20% present depressive symptoms [11]. The percentage of patients with psoriasis with depressive symptoms varies between 9% and 55% across different populations [12]. Another common condition among patients with psoriasis is the presence of anxiety symptoms [13]. The global prevalence of anxiety disorders in patients with psoriasis varies widely, ranging from 9% to 34% of individuals [14].

Patients with psoriasis in North America have shown a higher prevalence of depression and suicide, while those in South America presented a higher prevalence of anxiety [13]. The incidence of depression in adult patients with psoriasis was 42.1 per 1000 person-years, anxiety was 24.7, and the incidence of suicide was 2.6 per 1000 person-years [13]. Recent evidence reports that the prevalence of depression is 20%, that of anxiety is 21%, and that of suicide is 0.77% in adults with psoriasis [13]. Increased psoriasis severity is associated with lower quality of life and higher levels of anxiety and depression [15]. A prospective cohort study found that the severity of depression in patients with psoriasis escalates as psoriasis worsens [16]. Similarly, a meta-analysis indicates that deteriorating quality of life in patients with psoriasis is strongly linked to greater depression [17]. Moderate–severe psoriasis increases the likelihood of anxiety by 14%, while psoriatic arthritis doubles the risk of anxiety [17].

Despite the significant psychiatric burden among patients with psoriasis, research specifically addressing the intersection of psoriasis, depression, and anxiety remains limited. Existing bibliometric analyses predominantly focus on psoriasis as the disease itself, leaving gaps in our understanding of its psychosocial comorbidities and their research trends [18,19,20,21]. To bridge this gap, we conducted a bibliometric analysis that incorporates three database searches and employs advanced methodologies, including Latent Dirichlet Allocation (LDA) [22] and HJ-Biplot [23]. Our analysis offers an updated and comprehensive examination of research trends, focusing specifically on depression and anxiety in individuals with psoriasis. This study fills the gaps in existing bibliometric reviews by providing a unique and integrated understanding of the main psychiatric comorbidities associated with psoriasis and their impact on patients. To our knowledge, this is the first bibliometric analysis to specifically explore depression and anxiety in individuals with psoriasis. By identifying trends and key topics studied over the years, our analysis provides a valuable direction for future research in this field.

## 2. Materials and Methods

### 2.1. Study Design

The research focused on the following questions: (Q1) What are the main sources of publications and key contributors to psoriasis, depression, and anxiety research? (Q2) What scientific collaborations have emerged between countries in this field? (Q3) What are the dominant research topics within the study of psoriasis, depression, and anxiety? (Q4) How have these research topics evolved over time? (Q5) How are these topics distributed across scientific journals? 

Our study began with a systematic and reproducible approach to gather and refine a relevant sample of peer-reviewed scientific publications; then, we conducted a bibliometric analysis, applying a quantitative method to examine the academic literature, using bibliographies to provide detailed description, evaluation, and tracking of published research [24]. Next, we applied topic modeling with the Latent Dirichlet Allocation (LDA) algorithm [22], an unsupervised machine learning technique, to identify key research topics and their trends; in the following phase, we utilized the HJ-Biplot method [23], a multivariate statistical tool that enhances the analysis of the LDA model [25,26,27,28]. This method visualizes the data in a matrix, revealing the relationships between rows (entities) and columns (indicators) [23].

### 2.2. Data Collection

We employed a combination of renowned databases to ensure a comprehensive review of the relevant literature. Scopus and Web of Science (WoS) [29,30,31] were referred to; additionally, PubMed, overseen by the National Library of Medicine (NLM), emerged as a pivotal resource, particularly for the biomedical and life sciences literature. PubMed is recognized for its extensive coverage of articles from biomedical journals spanning clinical research, basic science, and public health disciplines. Leveraging PubMed alongside Scopus and Web of Science allowed us to access a diverse array of peer-reviewed articles, clinical trials, systematic reviews, and other invaluable sources, facilitating a comprehensive analysis of the literature germane to our study [32]. Integrating three datasets can present challenges, particularly due to the variability in article information depending on whether the source is Scopus, PubMed, or Web of Science. To address this, a method for merging the Scopus, PubMed, and Web of Science databases was replicated using the R 4.4.1 ‘Bibliometrix’ package 4.3.2 [33].

Supplementary Materials Table S1 presents the search strategies employed in three bibliographic databases: Scopus, Web of Science, and PubMed. These strategies were used to retrieve relevant articles on the topics of psoriasis, depression, and anxiety. Each database search included specific search strings designed to capture articles related to psoriasis and various aspects of depression, such as depressive disorders, mood depressive disorder, systematic reviews, clinical trials, and other relevant terms, equally for anxiety. The table provides details on the search date, search string used, and the number of results obtained from each database search. The initial search across the three databases yielded a total of 12,298 documents. Following the removal of duplicates, the database was reduced to 5142 records. Subsequently, documents lacking abstracts and affiliation were excluded, resulting in a final dataset of 5059 documents and papers published over the years from 1974 to 2023. The filtering process was applied to the initial document samples from PubMed, Scopus, and Web of Science. It showed the initial document counts from each database alongside the final number of documents after undergoing a thorough filtering process. This process excluded duplicates, misclassified entries, and documents without abstracts. The final sample included 5013 documents that met the specified inclusion criteria.

To ensure transparency and reproducibility, the selection and filtering criteria used in the data collection process are explicitly detailed in Section 2. This allows for the replication of the study and ensures that the results are verifiable. Additionally, ethical considerations related to data science and bibliometric research have been addressed, emphasizing the responsible management of bibliographic information. This includes ensuring that the analysis is conducted in an objective and unbiased manner, while maintaining the integrity and credibility of the research process.

#### 2.2.1. Bibliometrics Analysis

Bibliometrics, a quantitative method used for analyzing the academic literature through the examination of bibliographies, provides a way to describe, evaluate, and monitor published works. Various bibliometric techniques are tailored to specific research inquiries, allowing for scientific mapping by addressing common questions through bibliometric analysis [33]. In this study, we adopted an objective and reliable approach, considering four levels of analysis: countries, sources, documents, and authors. To facilitate this analysis, we utilized the bibliometrix R-Tool [33], an R package [34] that provides specialized tools for quantitative bibliometric and scientometric research.

#### 2.2.2. Topic Model

Topic modeling is a machine learning technique used to automatically discover (unsupervised) what topic or topics are present in a single document, or in a set of them. Topic modelling typically uncovers latent or hidden topics, topics that are not explicitly stated within the documents. Such latent topics are described by groups of words that one would commonly use to describe something, and such words typically occur within the same linguistic context [35].

To uncover latent topics, the topic model method Latent Dirichlet Allocation (LDA) was used; this is a method used within unsupervised text mining, in which document themes or topics can be identified from a larger collection of compiled documents known as a corpus. LDA is based on Bayesian models and is considered to be an extension of Probabilistic Latent Semantic Analysis [22,36]. The underlying idea of the LDA model is that the documents analyzed are represented by a random mix of latent topics and each topic in turn is characterized by a distribution of words that are the basic units that form the vocabulary [22]. The LDA model assumes that a fixed number of themes or categories are distributed over the entire collection of documents. Moreover, it is assumed that each document in the corpus addresses several themes and that each term is assigned a probability of belonging to a particular topic; thus, a document might be 60% about health and 40% about other uncovered topics. This is the topic probability distribution that can be inferred for each document. This method provides a robust framework for uncovering hidden patterns in complex datasets, making it highly valuable for analyzing research health areas.

#### 2.2.3. Identifying Research Topics

The procedure for the identification of topics through LDA was divided into three stages: (i) preprocessing, (ii) creation of LDA model, and (iii) labelling topics. Data processing in this part of the study was carried out using LDAshiny [22], an opensource package to R programming language [34]; this contains the development of a tool that provides a web-based graphical user interface to perform a review of the scientific literature under the Bayesian approach of Latent Dirichlet Allocation (LDA) and machine learning algorithms.

#### 2.2.4. Preprocessing Texts

A set of important preprocessing operations were carried out on the downloaded articles. Ref. [37] designates this phase as “text refining”, since the idea is to convert or transform the documents into an appropriate common format that allows the text to be structured for later analysis. To increase the coherence of the topics, each abstract was tokenized using bigrams which are the combination of consecutive unigrams. Although this process may seem trivial, the text downloaded to the computer must be converted to lowercase, and punctuation marks, dashes, brackets, numbers, spaces, and other characters must be removed. In addition, a standard list of words called “stopwords” was identified and eliminated, as these words (such as articles and prepositions) mainly serve to make a sentence grammatically correct.

#### 2.2.5. Creation Model Latent Dirichlet Allocation

Topic models are latent variable models of documents that use correlations between words and latent semantic themes in a collection of documents [38]. This definition assumes that the expected number of topics *k* (i.e., latent variables) must be established a priori. Thus, the selection process of the right number of topics for a given collection of articles is not trivial. This problem has been dealt with in different ways, but always considering the balance between the need to have a large number of topics (to cover all those in the document collection) and the need to have a limited number of topics, so that results are understandable. Simulations were carried out varying *k* from 5 to 40 in incremental steps of 1; an inference algorithm called Gibbs sampling, with 500 iterations, was used [39]. The quality LDA model was determined by utilizing a topic coherence measure [40], which is a measure of a topic model from the perspective of human interpretability and is considered a more adequate measure than computational metrics such as perplexity [41].

#### 2.2.6. Labelling Topics

The topics are not semantically labeled for the LDA model and algorithmic analyses are very limited in their ability to understand the latent meanings of human language, so manual labeling is considered a standard in topic modeling [42]. To provide a semantically correct interpretation, the topics were manually labeled using two sources of information: the most frequent word lists (most likely) and a sample of the titles. Then, summaries of the three most loaded articles were created. The topics identified by the LDA model require semantic labeling, as algorithmic approaches often struggle to fully capture the subtleties and contextual nuances of human language. As a result, manual labeling is widely recognized as the standard practice in topic modeling to ensure accurate interpretation and meaningful categorization [43]. 

#### 2.2.7. Quantitative Indices Used to Analyze the Trend of Topics

Due to the large number of articles and, therefore, the quantity of words, it is difficult to intuitively understand the topics and their trends. Therefore, we used some quantitative indices proposed by Xiong et al. [44], which are obtained by adding documents–topic and topic–words distributions in order to make the results and findings clear. The description of the indices is as follows. The distribution of topics over time is obtained by

(1) $$\theta_{k}^{y}=\frac{\sum_{m\epsilon y}\theta_{mk}}{n^{y}}$$

where mϵy represents articles published in a given year, $\theta_{mk}$ represents the proportion of the *k*-th topic in each item, and $n^{y}$ represents the total number of articles published in the year [44].

Topic distribution across journals is defined as the ratio of the *k*-th topic in the journal *j*: $\theta_{k}^{j}$

(2) $$\theta_{k}^{j}=\frac{\sum_{m\epsilon j}\theta_{mk}}{n^{j}}$$

where mϵj represents the articles in a particular journal, $\theta_{mk}$ represents the proportion of the *k*-th topic on each item, and $n^{j}$ represents the total number of articles published in the journal, *j*.

Topic distribution across countries is defined as the ratio of the *k*-th topic in the country, *c*:

(3) $$\theta_{k}^{c}=\frac{\sum_{m\epsilon c}\theta_{mk}}{n^{c}}$$

where *mϵc* represents the articles in a particular country, *θ_mk_* represents the proportion of the *k*-th topic on each item, and *n^c^* represents the total number of articles published in the country, *c*.

With the purpose of facilitating the characterization of the topics in terms of their tendency, we used simple regression slopes for each topic where the year was a dependent variable and the proportion of the topics in the corresponding year was the response variable [45]. The topics obtained by regression were positive or negative at a statistical significance level of 0.01 and were classified as positive or negative trends, respectively.

#### 2.2.8. HJ-Biplot

Biplots provide graphical representations of multivariate data, allowing for the visualization of three or more variables, much like a scatter plot does for two variables [46]. The HJ-Biplot [23] optimizes the simultaneous representation of both rows and columns in a low-dimensional space. It has proven particularly useful in enhancing the analysis of the Latent Dirichlet Allocation (LDA) model [25,26,27,28]. The distances between row markers signify their similarities, while the lengths of column markers (vectors) approximate the standard deviations. The cosines of the angles between column vectors provide an approximation of correlations, where acute angles suggest high positive correlation, obtuse angles indicate negative correlation, and right angles denote uncorrelated variables. The Multbiplot software 23.11.0 (2023-11-21) [47] was employed in this phase of the analysis.

In this study, we applied hierarchical cluster analysis to the initial dataset to uncover patterns and groupings among the variables. We used the Ward linkage method, which minimizes the sum of squared differences within clusters, effectively producing more homogeneous groups. This approach revealed natural groupings in the data, offering valuable insights into underlying relationships. This methodological choice was key to ensuring the robustness of our clustering results and improving the interpretability of the data structure.

## 3. Results

### 3.1. Bibliometrics Analysis

The analysis covers a time span from 1974 to 2023, during which 5059 documents have been published in 1151 different sources. The annual growth rate of publications in this field is 12.26%, indicating a significant increase in interest and research in this topic. The analyzed documents have an average age of 8.29 years and have received an average of 37.19 citations per document, reflecting the impact and relevance of this research in the scientific community.

The author analysis shows that a total of 18,686 authors have contributed to these studies, with 199 authors responsible for single-authored documents. Author collaboration is notable, with an average of 6.25 co-authors per document and 20.95% of publications resulting from international collaborations (Table 1).

**Figure 1:**
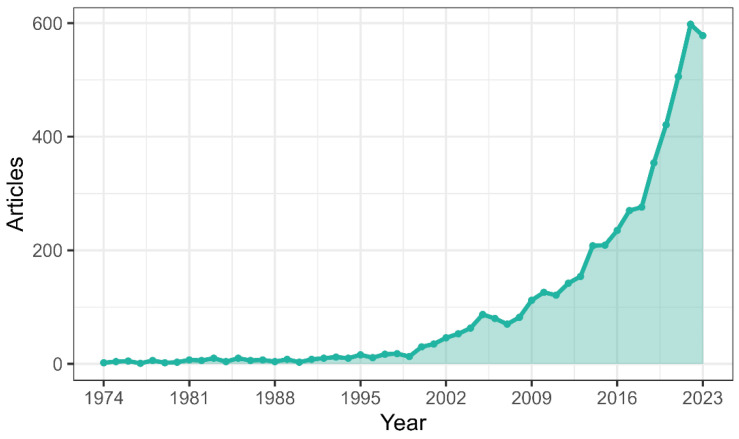
Annual scientific production about the collection of psoriasis from 5059, with articles published between 1974 and December 2023.

The analysis of annual scientific publication production on the relationship between depression and anxiety in psoriasis reveals several significant findings: During the early decades (1970s and 1980s), the number of publications was relatively low, fluctuating between 1 and 10 per year (Figure 1). From the 1990s onward, there has been a progressive increase in scientific production, with a notable rise in the number of publications per year. Growth accelerated in the early decades of the 21st century, marked by a significant uptick in annual publications. A turning point is highlighted in 2014, from which the number of publications experienced exponential growth. In recent years (2020 and 2021), unprecedented scientific production is recorded, with a notable increase in the number of publications, peaking in 2022 with 598 publications. Despite a slight decrease in 2023 to 578 publications, scientific production remains substantially high compared to earlier periods. These findings indicate continuous and significant growth in research on the relationship between depression, anxiety disorders, and psoriasis over recent decades, with a particularly notable increase in recent years. This suggests a growing interest and recognition of the importance of this area of study within the scientific community.

The top five journals in dermatological research have a significant impact in the field. *The Journal of the European Academy of Dermatology and Venereology* leads in the number of publications (251), with an h-index of 60 and 11,424 citations, establishing itself as a key source since 1994. *The British Journal of Dermatology*, founded in 1974, has the highest h-index (63) and 12,866 citations, standing out for its longevity and influence. *The Journal of Dermatological Treatment*, with 192 publications and an h-index of 33, focuses on dermatological therapies and has been relevant since 1989. *The Journal of the American Academy of Dermatology* combines a high h-index of 77 with 17,638 citations, reflecting its great influence since 1983, despite having 191 publications. Lastly, *Dermatologic Therapy*, though with fewer publications (127) and citations (2237), has an h-index of 26, highlighting its relevance in the field since its inception in 2002 (Table 2).

The five most prolific authors in the field have a significant impact. Feldman S.R. leads with 114 publications and an h-index of 36, accumulating 5799 citations since 1997, reflecting his lasting influence. Griffiths C.E.M., with 85 publications, has the highest h-index (42) and 7827 citations, positioning him as the most cited author since 1998. Puig L., with 74 publications and an h-index of 28, has been a key contributor since 1996, standing out for his consistent productivity. Armstrong A.W., with 69 publications since 2012 and an h-index of 31, has garnered 5973 citations, quickly establishing himself as a leading figure in recent research. Warren R.B., with 63 publications and an h-index of 26 since 2009, has accumulated 2202 citations, notable for his productivity in a shorter period. These authors play crucial roles in advancing knowledge in their field (Table 3). 

**Figure 2:**
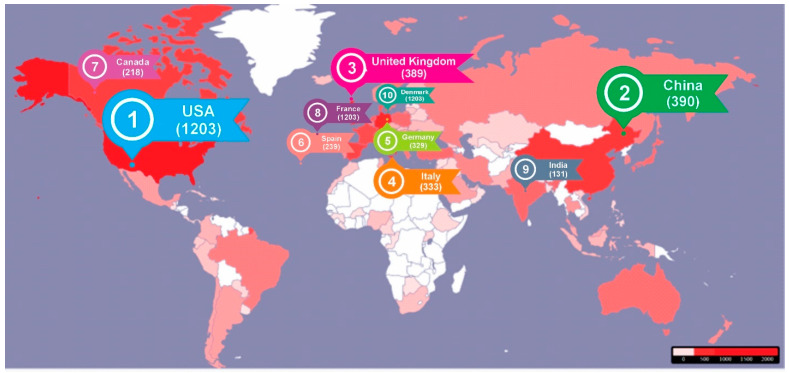
Distribution of geographical origins in the analysis of 5059 articles published from 1974 to 2023.

Figure 2 shows that contributions from 89 countries were identified regarding the research on the relationship between depression and anxiety disorders and psoriasis. The United States leads significantly with 1203 publications, representing approximately 25.8% of the total contributions. China ranks second with 390 publications. The United Kingdom and Italy also show a strong presence with 389 and 333 publications, respectively. Germany contributes 329 publications, ranking among the most active countries in this field of research. Other countries with notable contributions include Spain with 239 publications, Canada with 218 publications, France with 166 publications, India with 131 publications, and Denmark with 124 publications. Additionally, countries such as Turkey (123 publications), Poland (117 publications), and the Netherlands (106 publications) show considerable effort in researching this topic (Figure 2). The rest of the countries contribute fewer with less than 100 publications each, but collectively, they demonstrate a global effort in studying the relationship between psoriasis and anxiety and depression disorders. The geographical distribution of the publications indicates a greater contribution from developed countries, reflecting robust research capabilities and resources dedicated to studying the comorbidity between psoriasis and these mental health disorders. This geographical diversity in research suggests a growing global attention to the psychological impact of psoriasis.

Table 4 highlights the 28 most productive countries for corresponding authors in psoriasis research, providing details on the number of articles published, self-citation practices (SCP), multi-country collaborations (MCP), and the MCP ratio. This information offers valuable insights into each country’s research output and collaborative efforts in the field of psoriasis.

The five most productive countries in psoriasis research show varied approaches to collaboration. The United States leads with 1025 articles and a high proportion of international collaborations (26.93%), emphasizing its involvement in global networks. China, with 462 publications, focuses primarily on national research, with only 7.79% of international collaborations. Italy, with 353 articles, balances its approach, with 14.45% involving international partnerships. Germany and the United Kingdom stand out for their strong tendency toward international collaboration, with 32.55% and 32.65%, respectively, highlighting the importance of global cooperation in their research efforts.

**Figure 3:**
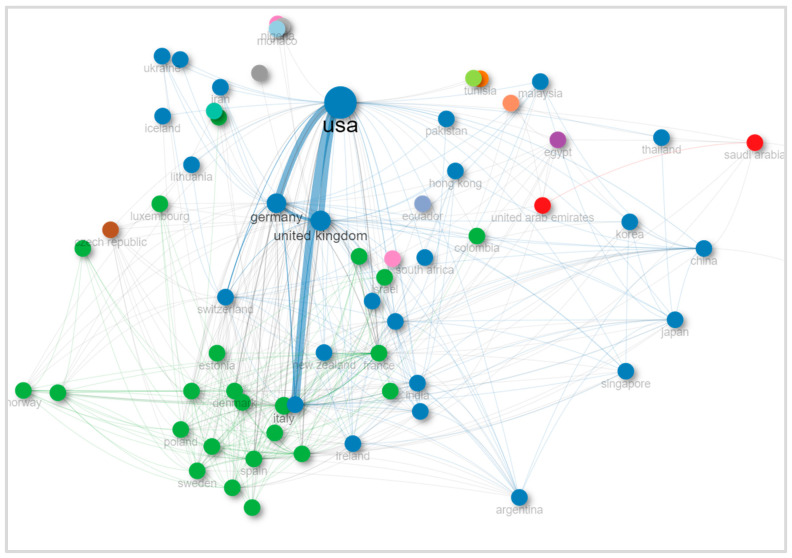
Collaboration network of countries on research about depression and anxiety in psoriasis patients.

The analysis of international collaboration in psoriasis research revealed important patterns (Figure 3). The United States (USA) emerged as the leading collaborator, while China also demonstrated strong partnerships. Italy, Germany, the United Kingdom, and Spain showed varying degrees of collaboration in rates of our study.

### 3.2. Latent Dirichlet Allocation

The LDA model, which achieved the optimal coherence score, comprises 28 topics (*k* = 28). Table 5 presents the 15 most probable terms (i.e., the terms with the highest probabilities) for each latent topic, along with a semantically related label. Each topic is characterized by its prevalence in the corpus, the most relevant terms, a descriptive label, and the number of documents (N).

Table 5 provides a comprehensive and structured insight into current dermatological research, addressing not only treatment efficacy and comorbidity management, but also the psychological and emotional impact of skin diseases on patients. This holistic approach is crucial for enhancing both clinical management and quality of life for individuals affected by dermatological conditions. Table 5 highlights significant research at the intersection of mental health and dermatology. It identifies 430 studies exploring the relationship between depression, anxiety, and skin diseases, emphasizing how these psychological disorders affect patients’ quality of life. Additionally, key areas of investigation include 232 studies on symptom assessment and quality of life in dermatological patients, using tools like the Dermatology Life Quality Index (DLQI) to measure the impact of symptoms on daily life. The data also reveal a focus on clinical and therapeutic research, with 1007 studies evaluating the effectiveness of treatments such as plaquenil and placebo in patients with moderate severity. These studies are essential for understanding how treatments impact both the physical and mental health of patients.

### 3.3. Topic Trends

**Figure 4:**
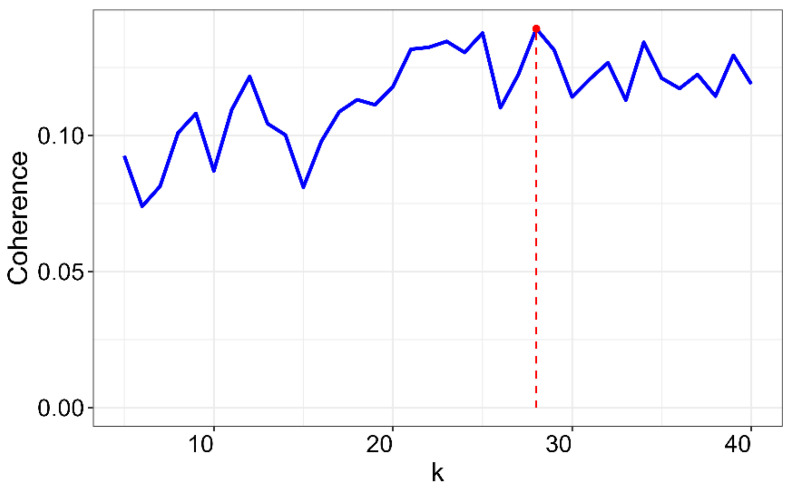
Coherence.

The document–topic distribution, represented as *θ_m_*, was used to compute the mean probability $\theta_{k}^{y}$ of articles published in a given year to identify emerging trends, as shown in Figure 4. The analysis highlighted certain topics with steadily increasing probabilities over time, indicated in red. In contrast, topics with declining probabilities are marked in blue, and those without a clear trend and that maintained stable over time are shown in black.

#### HJ-Biplot

**Figure 5:**
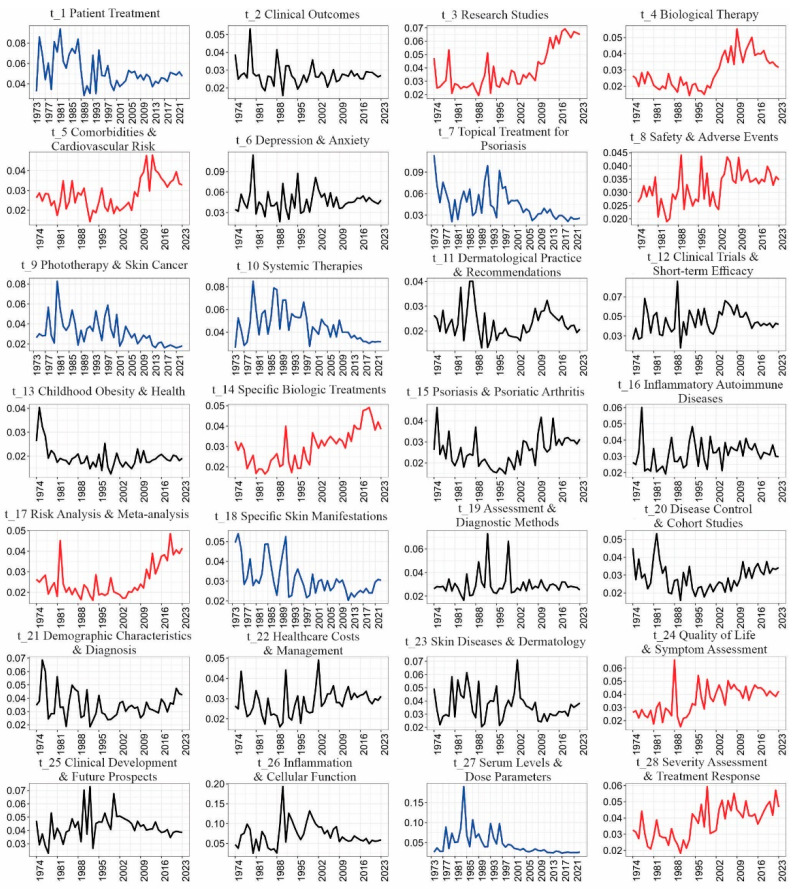
Diagrams of topic trends.

The analysis was performed across temporal, periodical, and geographical dimensions using multivariate HJ-Biplot analysis, which produced theta matrix outputs. The probability coefficients for each matrix ranged from 0 to 1. As shown in Figure 4, the percentages of data variability at the intersections of topics with years (57.88%), topics with countries (62.72%), and topics with journals (51.14%) were calculated, revealing stable clusters, as depicted in Figure 5.

**Figure 6:**
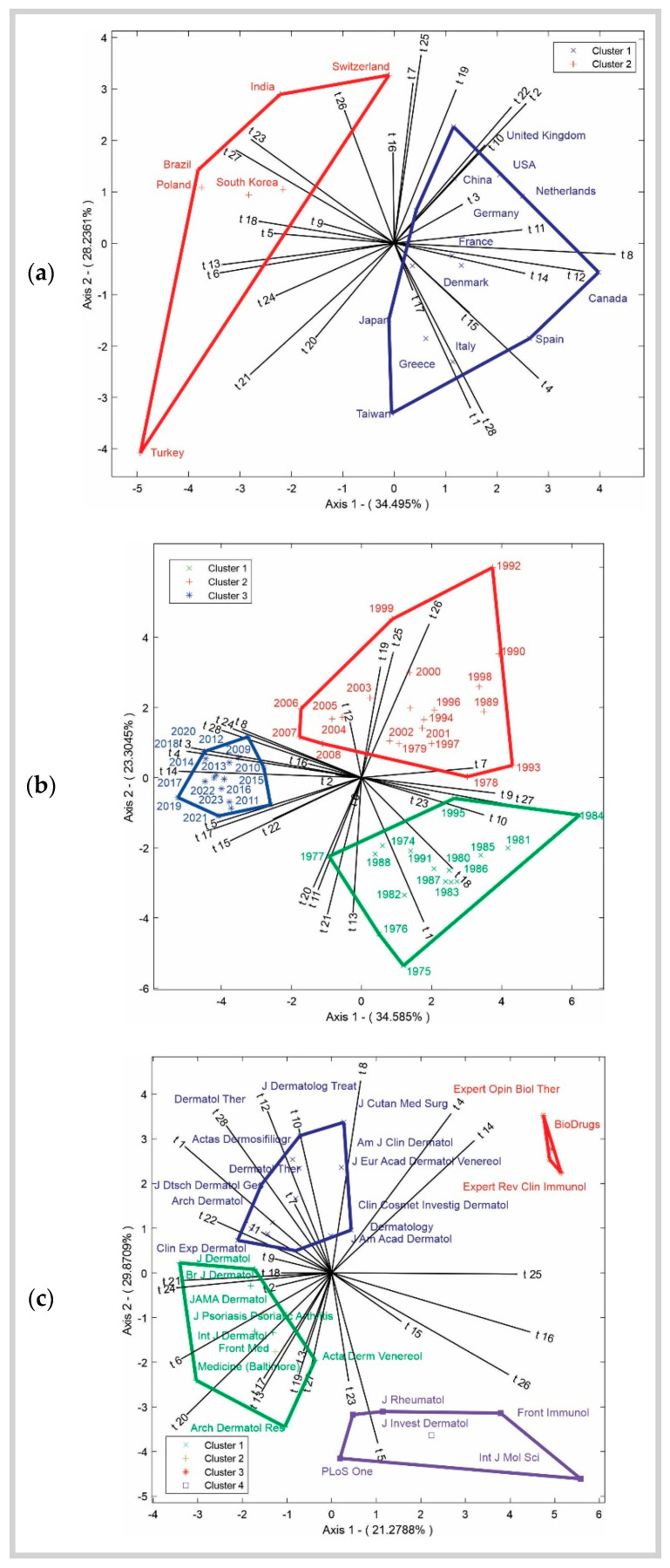
Association among topics per country (**a**), topics per year (**b**), and topics per journal (**c**) using the HJ-Biplot method.

In Figure 6a, the red group shows that Turkey, India, Switzerland, Brazil, Poland, and South Korea are positively related to topics t_5 (Comorbidities and Cardiovascular Risk), t_6 (Depression and Anxiety), t_9 (Phototherapy and Skin Cancer), t_13 (Childhood Obesity and Health), t_16 (Inflammatory Autoimmune Diseases), t_18 (Risk Analysis and Meta-analysis Specific Skin Manifestations), t_23 (Skin Diseases and Dermatology), t_20 (Disease Control and Cohort Studies), t_21 (Demographic Characteristics and Diagnosis), t_24 (Quality of Life and Symptom Assessment), t_26 (Inflammation and Cellular Function), and t_27 (Serum Levels and Dose Parameters). In the blue group, the countries United States, United Kingdom, China, Germany, France, Canada, Japan, Greece, Italy, Spain, Taiwan, Netherlands, and Denmark are directly linked to topics t_7 (Topical Treatment for Psoriasis), t_25 (Serum Levels and Dose Parameters), t_19 (Assessment and Diagnostic Methods), t_22 (Healthcare Costs and Management), t_10 (Systemic Therapies), t_22 (Healthcare Costs and Management), t_2 (Clinical Outcomes), t_3 (Research Studies), t_11 (Dermatological Practice and Recommendations), t_8 (Safety and Adverse Events), t_12 (Clinical Trials and Short-term Efficacy), t_14 (Specific Biologic Treatments), t_4 (Biological Therapy), t_15 (Psoriasis and Psoriatic Arthritis), t_28 (Severity Assessment and Treatment Response), t_1 (Patient Treatment), and t_17 (Risk Analysis and Meta-analysis). These relationships suggest that the countries share characteristics based on the closest topics, which represent the key variables explaining their grouping.

In Figure 6b, the blue group, which includes the most recent years such as 2020, 2021, 2022, and 2023, clusters around topics t_4 (Biological Therapy), t_8 (Safety and Adverse Events), t_24 (Quality of Life and Symptom Assessment), t_16 (Inflammatory Autoimmune Diseases), t_2 (Clinical Outcomes), t_5 (Comorbidities and Cardiovascular Risk), t_14 (Specific Biologic Treatments), t_17 (Risk Analysis and Meta-analysis), t_15 (Psoriasis and Psoriatic Arthritis), t_22 (Healthcare Costs and Management), t_3 (Research Studies), t_28 (Severity Assessment and Treatment Response), and t_24 (Quality of Life and Symptom Assessment) are positioned closely together. The green group, covering the years between 1974 and 1995, shows positive associations with topics t_9 (Phototherapy and Skin Cancer), t_27 (Serum Levels and Dose Parameters), t_10 (Systemic Therapies), t_23 (Skin Diseases and Dermatology), t_18 (Specific Skin Manifestations), t_1 (Patient Treatment), t_13 (Childhood Obesity and Health), t_21 (Demographic Characteristics and Diagnosis), t_11 (Dermatological Practice and Recommendations), t_20 (Disease Control and Cohort Studies), and t_21 (Demographic Characteristics and Diagnosis). This suggests that, during the 1980s and early 1990s, these topics played a significant role in the research or developments of that time, highlighting a clear thematic evolution throughout the years in this cluster. The red group, spanning from 1979 to 2008, displays a strong positive relationship with topics t_7 (Topical Treatment for Psoriasis), t_9 (Phototherapy and Skin Cancer), t_12 (Clinical Trials and Short-term Efficacy), t_19 (Assessment and Diagnostic Methods), t_25 (Clinical Development and Future Prospects), and t_26 (Inflammation and Cellular Function).

Finally, in Figure 6c, the journals in the green group include *J Dermatol*, *Br J Dermatol*, *JAMA Dermatol*, *J Psoriasis Psoria Arthritis*, *Int J Dermatol*, *Front Med*, *Acta Derm Venereol*, *Medicine* (Baltimore), and *Arch Dermatol Res*. These journals are strongly associated with topics t_18 (Specific Skin Manifestations), t_21 (Demographic Characteristics and Diagnosis), t_24 (Quality of Life and Symptom Assessment), t_6 (Depression and Anxiety), t_17 (Risk Analysis and Meta-analysis), t_13 (Childhood Obesity and Health), t_1 (Patient Treatment), t_9 (Phototherapy and Skin Cancer), t_3 (Research Studies), t_19 (Assessment and Diagnostic Methods), t_27 (Serum Levels and Dose Parameters), and t_20 (Disease Control and Cohort Studies). The purple group includes journals like *J Invest Dermatol*, *J Rheumatol*, *Front Immunol*, *Int J Mol Sci*, and *PLoS One*, which are related to topics t_23 (Skin Diseases and Dermatology), t_5 (Comorbidities and Cardiovascular Risk), t_15 (Psoriasis and Psoriatic Arthritis), t_26 (Inflammation and Cellular Function), t_16 (Inflammatory Autoimmune Diseases), and t_25 (Clinical Development and Future Prospects). The red group consists of journals such as *Expert Opin Biol Ther*, *Expert Rev Clin Immunol*, and *BioDrugs*, which are strongly associated with topics t_4 and t_14. The blue cluster includes journals like *Clin Exp Dermatol*, *Arch Dermatol*, *J Dtsch Dermatol Ges*, *Dermatol Ther*, *Acta Dermosifiliogr*, *Dermatol Ther*, *J Dermatolog Treat*, *J Cutan Med Surg*, *Am J Clin Dermatol*, *J Eur Acad Dermatol Venereol*, *Clin Cosmet Investig Dermatol*, *Dermatology*, and *J Am Acad Dermatol*, which are closely related to topics t_22 (Healthcare Costs and Management), t_11 (Dermatological Practice and Recommendations), t_1 (Patient Treatment), t_28 (Severity Assessment and Treatment Response), t_12 (Clinical Trials and Short-term Efficacy), t_10 (Systemic Therapies), and t_8 (Safety and Adverse Events). In summary, in Figure 6a, the clustering of countries suggests distinct research priorities, with some nations focusing on clinical and therapeutic aspects while others emphasize epidemiological and socioeconomic factors. Figure 6b highlights the temporal evolution of research trends, demonstrating a shift from early studies on phototherapy and systemic therapies to more recent investigations into biological treatments, quality of life, and healthcare costs. Figure 6c illustrates the role of specific journals in disseminating research on different thematic areas, with some journals predominantly publishing studies on dermatological practice. In contrast, others focus on immunological and molecular aspects. These patterns reflect the dynamic progression of psoriasis research and the field’s interdisciplinary nature.

## 4. Discussion

This bibliometric analysis reveals a total of 5059 published documents on the research of psoriasis and its relationship with depression and anxiety across 1151 sources. During the 49 years, a steady increase in research output about psoriasis, depression, and anxiety over time is suggested by the annual growth rate of 12.26% observed in the results of our study. The leading journals are the Journal of the European Academy of Dermatology and Venereology and the British Journal of Dermatology. Predictably, the United States is the most contributing nation in terms of research on psoriasis, depression, and anxiety. China ranks second on the contribution of this research theme, followed by Italy and Germany. The United States and China lead collaborative research on the disease, with the most actively engaged writers.

Several vital topics emerged from the 5059 articles on psoriasis published between 1974 and 2023. The five most prevalent research topics were inflammation and cellular function, followed by research studies, depression and anxiety, severity assessment and treatment response, and clinical trials and short-term efficacy. Quality of life and symptom assessment was also a significant area of focus in psoriasis research. This study specifically aims to investigate the impact of depression and anxiety on patients with psoriasis. The topics published over the past 49 years reflect a growing concern about the comorbidity of depression and anxiety in patients with psoriasis, and their poor impact on patients’ quality of life.

### 4.1. Interpretation of Principal Findings

The worldwide psoriasis epidemiology reveals rates of 0.51% to 11.43% in adults and 0% to 1.37% in children [48]. The prevalence of psoriasis in persons in the United States aged 20 or older is 3.0%, with a particularly greater occurrence in white individuals [49]. In pediatric patients, the prevalence is 9.3 per 100,000, with higher rates in American people aged 15–17 years [50]. In our study, the United States leads in publications on the disease, followed by China, Italy, and Germany. Our results corroborate a previous bibliometric review where the United States ranked as the leading country on research about psoriasis [18]. It is worth mentioning that our results include psychiatric disorders such as depression and anxiety in patients with psoriasis. The association between psoriasis and depression is well-established in the population of the United States [51]. The incidence of psoriasis is significantly higher in the United States than in China, at 2–4% and 0.47%, respectively [52,53].

Our results show the leading journals that provide information about psoriasis, depression, and anxiety. The three firstly were following: *The Journal of the European Academy of Dermatology and Venereology*, which has an h-index of 60; *The British Journal of Dermatology*, which leads with 63; and *The Journal of Dermatological Treatment*, which holds an h-index of 33. Previous bibliometric research assessed the relationship between psoriasis and endothelial cells, and the *Journal of Investigative Dermatology* is considered the most influential journal in this field [20,21]. Our analysis shows a yearly rise in scientific interest in depression and anxiety in patients with psoriasis, as indicated by the increasing number of related publications. This trend highlights the growing focus of dermatology journals on the mental health of patients with psoriasis, emphasizing the importance of integrated care.

Our findings show that the most prevalent topic was “inflammation and cellular function”, while the research topic of “depression and anxiety” ranked third in prevalence during the last 49 years in psoriasis research. A systematic review reveals that patients with psoriasis are significantly more likely to experience depressive symptoms and clinical depression [11]. The comorbidity of psoriasis, depression, and anxiety is a critical issue that significantly impacts patients’ health [11]. The risk of depression is significantly higher in individuals with psoriasis compared to the general population [54]. Several hypotheses may explain the greater prevalence of depression and anxiety in patients with psoriasis. In observational studies, the location of injuries and the severity of the illness are related to depression and anxiety relief [16,17,55,56]. The biological link of psoriasis with mental well-being is hypothesized to be the hypothalamic–pituitary–adrenal (HPA) axis, which is deregulated in times of stress as a result of the increased proinflammatory mediators in psoriasis [8]. Furthermore, depression can cause an increase in inflammatory cytokines, the presence of chronic inflammation, and deregulate the immune system [8].

Psoriasis and comorbid depression lead to inflammatory mechanisms, mainly the increase in interleukins and cytokines [57,58]. This production of proinflammatory cytokines is associated with the overactivation of the HPA axis and with the positive regulation of serotonin transporters (5-HTT) [7]. Serotonin, a key neurotransmitter in depression, can be reduced in neurons due to the positive regulation of 5-HTT [59,60]. Interleukins have been observed to be among the most commonly implicated inflammatory markers in the pathogenesis of both depression and psoriasis [7]. For example, patients with psoriasis and comorbid depression have shown elevated serum concentrations of IL-6, IL-18, and IL-17A [58]. Additionally, bidirectional mendelian randomization analyses have shown a genetic risk of psoriasis being positively associated with depressive disorders, which might indicate possible causal relationships [61,62]. These findings highlight the importance of screening for depression in patients suffering from psoriasis [61,62]. Accepted hypotheses suggest that increased sympathetic activity, proinflammatory mediator release, and HPA axis hyperactivation are potential associations between psoriasis and psychiatric diseases [62]. However, immunological hypotheses have currently suggested a possible immuno-genetic link between mental health disorders and psoriasis [9,10]. The polygenic susceptibility to immunological abnormalities in patients with psychiatric disorders may overlap with psoriasis inheritance [9,10].

Inflammation and cellular function emerged as the most prominent topic in psoriasis bibliometric research. It is worth highlighting that specific biological treatments ranked seventh and biological therapies ranked eighth among the identified topics. Despite significant advancements in understanding the biological link between psoriasis, depression, and anxiety, there is still a lack of translation of these findings into clinical applications. Randomized controlled trials (RCTs) highlight the short-term benefits of biological therapies in improving depression and anxiety symptoms in patients with psoriasis [17]. Perhaps future clinical trial studies can underscore the use of therapeutic targeting of systemic inflammation to achieve dermatological and psychiatric improvements.

Our results indicate a higher interest among research studies in focusing on depression and anxiety, severity assessment, and treatment response in terms of short-term efficacy. The research topic of “Quality of Life and Symptom Assessment” ranked sixth among the most prevalent topics, with evaluations frequently based on short-term studies. Future studies should focus on patient-reported outcomes in long-term follow-ups to assess improvements in quality of life and mental health. This approach allows for the evaluation of the durability of biologic therapies and guiding patient-centered care strategies. Our results partially observed this tendency, as topics like Disease Control and Cohort Studies ranked lower (23rd among the 28 identified hotspots). Future research efforts should address unanswered questions about the sustained impacts of biologic therapies on mental health outcomes in patients with psoriasis over extended periods.

Analysis of the results of various clinical trials has led to the hypothesis that the effects of anti-inflammatory drugs on depressive symptoms can not only attributed to the alleviation of physical health for the primary disease [63]. In fact, the antidepressant effect of some anti-inflammatory drugs such as ustekinumab (an anti-IL-12/23 antibody) was significant after adjusting for physical health outcomes, and the effect of sirukumab and siltuximab (anti-IL-6 antibodies) were significant in non-responders for the primary disease [63]. Depressive and anxiety symptoms in psoriasis seem to be related to inflammation, making the use of anti-inflammatory drugs a potential strategy not only for improving physical health in relation to the primary disease but also for enhancing the mental health of these patients [64]. The use of IL-23p19 inhibitors such as Risankizumab [65] and guselkumab [66] has been shown to lead to significant improvements in anxiety and depressive symptoms for patients with psoriasis. Likewise, anti-IL-17A antibodies such as Secukinumab [67] and Ixekizumab [68] have been observed to improve evaluated anxiety and depression in psoriasis. On the other hand, anti-TNF drugs have been shown to have a less pronounced effect on these mental comorbidities in psoriasis, with guselkumab being superior to the TNF-antagonist adalimumab [69].

### 4.2. Limitations and Strengths of the Study

Some limitations of this study are important to consider. The reliance on English-language publications may exclude significant contributions from non-English sources, potentially underrepresenting research from regions where English is not the primary academic language. Future studies could address this limitation by incorporating multilingual databases. Furthermore, the word search focused on a limited number of keywords related to depression, anxiety, and psoriasis, leading to the possibility of missing scientific papers with varied disorder definitions and providing in false-negative results. Limiting the analysis to a specific year range excludes research that was published outside those years; thus, caution is advised when interpreting the results. Although this study identifies differences in publication output among countries, it does not statistically analyze these discrepancies. Future research should incorporate statistical approaches to understand regional variations in scientific contributions and their potential influencing factors better. Nevertheless, this study provides a valuable landscape of the research trends on psychiatric comorbidities in psoriasis. To the best of our knowledge, this is the first bibliometric analysis examining depression and anxiety in individuals with psoriasis. One strength of this study is the use of three databases (PubMed, Scopus, and Web of Science); these extensive data allow for a comprehensive analysis to be conducted on the evolution of research on depression, anxiety, and psoriasis. Advanced statistical methods, such as LDA and HJ-Biplot, improved the robustness of the findings of our study.

## 5. Conclusions

This bibliometric analysis highlights significant trends in research on psoriasis, depression, and anxiety, revealing a steady growth in publications, with an annual increase of 12.26%. The United States, China, Italy, and Germany emerge as leading contributors, reflecting a global commitment to understanding these comorbidities. Our findings emphasize the critical link between psoriasis, depression, and anxiety, which ranked as the third most crucial research topic over the years of publication studied. From a clinical perspective, bibliometric findings underscore the need to integrate care strategies that address the dermatological and psychiatric aspects of psoriasis.

With psoriasis, patients need routine mental health screenings, coupled with treatment protocols that simultaneously target inflammation and mental health. Biological therapies may be an appropriate option for dermatological efficacy and for alleviating mental health burdens, comprising long-term, patient-centered solutions. Finally, further exploration of the shared pathophysiological mechanisms between psoriasis and psychiatric disorders is essential if we are to deepen our understanding of their comorbidity and to guide therapeutic innovations. Future research should center gaps in the long-term outcomes of therapies for clinical practice and for improving the mental health of patients with psoriasis.

## Figures and Tables

**Table 1:** Main information about bibliometric analysis of research on psoriasis.

Description	Results
MAIN INFORMATION ABOUT DATA	
Timespan	1974:2023
Sources (journals)	1151
Documents	5059
Annual growth rate, %	12.26
Document average age	8.29
Average citations per doc	37.19
References	6576
DOCUMENT CONTENTS	
Keywords plus (ID)	16,808
Author’s keywords (DE)	6272
AUTHORS	
Authors	18,686
Authors of single-authored docs	199
AUTHORS COLLABORATION	
Single-authored docs	232
Co-authors per doc	6.25
International co-authorships %	20.95

**Table 2:** Top 30 scientific journals for research on psoriasis, h-index, TC = total citations, NP = number of publications, and PY_start = year of publication start. The table is organized in descending order by NP.

Source	h_Index	TC	NP	PY_Start
*Journal of The European Academy of Dermatology and Venereology*	60	11,424	251	1994
*British Journal of Dermatology*	63	12,866	218	1974
*Journal of Dermatological Treatment*	33	3731	192	1989
*Journal of The American Academy of Dermatology*	77	17,638	191	1983
*Dermatologic Therapy*	26	2237	127	2002
*Dermatology And Therapy*	20	1344	105	2012
*American Journal of Clinical Dermatology*	33	3554	85	2001
*Dermatology*	28	2627	78	1977
*Acta Dermato-Venereologica*	31	2756	75	2002
*Journal of Dermatology*	21	1741	69	1994
*International Journal of Dermatology*	26	2231	67	1981
*Journal of Cutaneous Medicine and Surgery*	19	991	57	1997
*Jama Dermatology*	32	3602	56	2013
*Archives of Dermatological Research*	22	1153	55	1982
*Actas Dermo-Sifiliograficas*	15	638	55	2002
*Journal of Rheumatology*	22	1608	49	2009
*Journal of Investigative Dermatology*	34	6407	48	1978
*Clinical And Experimental Dermatology*	18	881	45	1978
*Expert Review of Clinical Immunology*	17	830	45	2008
*Expert Opinion on Biological Therapy*	17	916	38	2001
*Plos One*	18	1229	37	2007
*Frontiers In Immunology*	15	1447	35	2013
*Jddg—Journal of The German Society of Dermatology*	14	739	35	2004
*Frontiers In Medicine*	10	342	34	2020
*Medicine* (United States)	9	407	34	2015
*International Journal of Molecular Sciences*	17	1062	33	2015
*Clinical, Cosmetic and Investigational Dermatology*	12	672	33	2009
*Archives Of Dermatology*	22	2456	28	1978
*Biodrugs*	18	1094	28	1997
*Journal Of Psoriasis and Psoriatic Arthritis*	6	125	27	2018

**Table 3:** Top 10 most prolific authors in psoriasis. NP = number of publications, h-index, TC = total citations. The table is organized in descending order by NP.

Author	h_Index	TC	NP	PY_Start
FELDMAN, S.R.	36	5799	114	1997
GRIFFITHS, C.E.M.	42	7827	85	1998
PUIG, L.	28	3035	74	1996
ARMSTRONG, A.W.	31	5973	69	2012
WARREN, R.B.	26	2202	63	2009
REICH, K.	31	3176	56	2001
GOTTLIEB, A.B.	27	3113	56	2000
WU, J.J.	23	2194	56	1998
AUGUSTIN, M.	25	2185	53	2005
PAUL, C.	27	2992	51	2009
MENTER, A.	27	3508	50	2000
LEBWOHL, M.	27	2415	46	1998
SKOV, L.	23	2156	44	2003
KIMBALL, A.B.	27	3750	43	2004
MROWIETZ, U.	21	1664	39	2002
MEGNA, M.	18	771	39	2017
PAPP, K.A.	19	1376	38	2004
EGEBERG, A.	17	1284	37	2015
BIANCHI, L.	16	596	37	2014
WANG, Y.	12	559	37	2013

**Table 4:** Top 20 most productive countries of corresponding authors on psoriasis.

Country	Articles	SCP	MCP	MCP %
USA	1025	749	276	26.9268293
CHINA	462	426	36	7.79220779
ITALY	353	302	51	14.4475921
GERMANY	298	201	97	32.5503356
UNITED KINGDOM	291	196	95	32.6460481
SPAIN	217	198	19	8.75576037
CANADA	190	124	66	34.7368421
FRANCE	159	116	43	27.0440252
INDIA	119	112	7	5.88235294
TURKEY	114	113	1	0.87719298
DENMARK	106	69	37	34.9056604
POLAND	102	90	12	11.7647059
NETHERLANDS	88	65	23	26.1363636
JAPAN	80	73	7	8.75
GREECE	65	53	12	18.4615385
KOREA	59	54	5	8.47457627
BRAZIL	55	47	8	14.5454545
SWITZERLAND	55	22	33	60
IRAN	54	38	16	29.6296296
AUSTRALIA	47	30	17	36.1702128

**Table 5:** Topics discovered from 5059 articles published on psoriasis between 1974 and 2023. N = number of publications.

T	Prevalence	Top_Terms	Label	N
t_1	4752	patient, month, treat, receiv, observ, treatment, retrospect, initi, rate, patient_treat, follow, period, time, discontinu, conclus	Patient Treatment	195
t_2	2707	outcom, trial, measur, clinic, particip, assess, intervent, compar, design, outcom_measur, primari, differ, clinic_trial, global, main	Clinical Outcomes	46
t_3	5795	studi, review, systemat, includ, search, evid, literatur, systemat_review, report, databas, control, data, identifi, pubm, rct	Research Studies	503
t_4	3655	biolog, tnf, agent, anti, treatment, drug, adalimumab, etanercept, infliximab, therapi, α, biolog_therapi, inhibitor, tnf_α, factor	Biological Therapy	218
t_5	3417	comorbid, risk, diseas, factor, cardiovascular, increas, diabet, risk_factor, preval, psoriat, metabol, patient, syndrom, popul, cardiovascular_diseas	Comorbidities and Cardiovascular Risk	206
t_6	4733	depress, anxieti, patient, psycholog, disord, symptom, stress, anxieti_depress, sever, psychiatr, mental, level, social, scale, suicid	Depression and Anxiety	430
t_7	2935	topic, treatment, effect, vulgari, daili, calcipotriol, mild, corticosteroid, combin, trial, clinic, scalp, chines, ointment, efficaci	Topical Treatment for Psoriasis	157
t_8	3533	safeti, advers, event, term, efficaci, advers_event, data, infect, trial, report, treatment, profil, efficaci_safeti, apremilast, rate	Safety and Adverse Events	111
t_9	2074	phototherapi, treatment, cancer, uvb, puva, therapi, nb, tuberculosi, nb_uvb, ultraviolet, light, treat, modal, skin, melanoma	Phototherapy and Skin Cancer	82
t_10	3519	therapi, treatment, effect, system, combin, methotrex, option, oral, convent, system_therapi, cyclosporin, agent, acitretin, system_treatment, treatment_option	Systemic Therapies	118
t_11	2282	treatment, dermatologi, academi, practic, academi_dermatologi, european, recommend, venereologi, dermatologi_venereologi, adher, european_academi, clinic_practic, satisfact, base, american	Dermatological Practice and Recommendations	29
t_12	4439	week, sever, moder, plaqu, placebo, moder_sever, patient, improv, trial, dose, random, efficaci, patient_moder, receiv, compar	Clinical Trials and Short-term Efficacy	237
t_13	1892	children, sleep, bodi, weight, adult, obes, pediatr, ag, p, bmi, loss, mass, popul, bodi_mass, adolesc	Childhood Obesity and Health	24
t_14	3925	secukinumab, trial, clinic, efficaci, clinic_trial, approv, treatment, antibodi, phase, ixekizumab, ustekinumab, inhibitor, interleukin, drug, safeti	Specific Biologic Treatments	229
t_15	2.99	psoriat, psa, arthriti, psoriat_arthriti, diseas, activ, joint, patient, arthriti_psa, diseas_activ, patient_psa, psa_patient, enthes, modifi, rheumatologi	Psoriasis and Psoriatic Arthritis	161
t_16	3269	diseas, inflammatori, autoimmun, arthriti, rheumatoid, rheumatoid_arthriti, ra, autoimmun_diseas, immun, inflammatori_diseas, bowel, inflammatori_bowel, bowel_diseas, immun_mediat, includ	Inflammatory Autoimmune Diseases	128
t_17	3501	risk, analysi, meta, meta_analysi, studi, increas, associ, ratio, interv, pool, confid, rr, incid, confid_interv, increas_risk	Risk Analysis and Meta-analysis	158
t_18	2725	nail, involv, clinic, lesion, onset, report, gpp, rare, featur, pustular, gener, diseas, flare, women, manifest	Specific Skin Manifestations	113
t_19	2821	assess, valid, method, dermatologist, measur, evalu, predict, correl, british, tool, identifi, specif, perform, biomark, associ_dermatologist	Assessment and Diagnostic Methods	76
t_20	3169	control, patient, compar, pso, adjust, cohort, differ, healthi, subject, rate, regress, ag, ratio, conclus, popul	Disease Control and Cohort Studies	93
t_21	3694	patient, hospit, ag, diagnosi, medic, infect, femal, covid, male, diagnos, data, characterist, record, aim, conclus	Demographic Characteristics and Diagnosis	176
t_22	3041	cost, medic, care, health, manag, data, base, util, effect, burden, cost_effect, healthcar, estim, econom, time	Healthcare Costs and Management	100
t_23	3408	skin, diseas, skin_diseas, condit, dermat, dermatolog, atop, disord, chronic, atop_dermat, inflammatori_skin, common, vitiligo, eczema, acn	Skin Diseases and Dermatology	153
t_24	4.13	life, qualiti, patient, qualiti_life, score, sever, dlqi, impact, symptom, improv, assess, dermatologi, report, life_qualiti, scale	Quality of Life and Symptom Assessment	232
t_25	4055	clinic, develop, trial, clinic_trial, therapeut, review, drug, discuss, current, approach, provid, potenti, futur, base, understand	Clinical Development and Future Prospects	112
t_26	6185	cell, inflammatori, activ, role, cytokin, target, immun, inflamm, mechan, pathwai, potenti, inhibit, receptor, express, pathogenesi	Inflammation and Cellular Function	601
t_27	2823	level, dose, increas, serum, dai, decreas, blood, liver, acid, concentr, mtx, drug, lesion, paramet, reduc	Serum Levels and Dose Parameters	96
t_28	4529	pasi, sever, respons, score, week, achiev, treatment, baselin, sever_pasi, clinic, patient, improv, pasi_score, evalu, effect	Severity Assessment and Treatment Response	275

## Data Availability

Data may be made available upon request.

## Supplementary Materials

The following supporting information can be downloaded at: https://www.mdpi.com/article/10.3390/healthcare13050441/s1, Table S1: Bibliographic Database Search Strategies.
