# Supplementary material for: Depression and Anxiety in Patients with Psoriasis: A Comprehensive Analysis Combining Bibliometrics, Latent Dirichlet Allocation, and HJ-Biplot
Source: Healthcare (Basel). 2025 Feb 20;13(5):441. doi: 10.3390/healthcare13050441 (PMC11899133; doi:10.3390/healthcare13050441)
Supplement: Supplementary file 1 [file healthcare-13-00441-s001.zip › healthcare-3377717-supplementary.pdf]

Supplementary Table S1. Bibliographic Database Search Strategies.

Bibliographic database	Search data	Search string	Results
Scopus	June 6, 2024	TITLE-ABS (psoriasis) AND (TITLE-ABS (depression) OR TITLE-ABS ("depressive disorders") OR TITLE-ABS ("mood depressive disorder") OR TITLE-ABS ("Symptom, major depressive disorder") OR TITLE-ABS ("Systematic Review") OR TITLE-ABS ("Systematic Review and meta-analysis") OR TITLE-ABS ("Clinical trial") OR TITLE-ABS ("Randomized Clinical Trial") OR TITLE-ABS ("Cross-section study") OR TITLE-ABS ("Longitudinal study") OR TITLE-ABS ("Retrospective study") OR TITLE-ABS ("Hospitalization") OR TITLE-ABS (psychotherapy) OR TITLE-ABS ("SSRIs") OR TITLE-ABS ("tricyclic antidepressant") OR TITLE-ABS ("Antidepressant therapy") OR TITLE-ABS ("cognitive behavioral therapy") OR TITLE-ABS ("non-pharmacological strategies") OR TITLE-ABS (anxiety) OR TITLE-ABS ("anxiety disorders")) AND PUBYEAR > 1973 AND PUBYEAR < 2024 AND (LIMIT-TO (DOCTYPE , "ar") OR LIMIT-TO (DOCTYPE , "re"))	N= 5,696
Web of Science	June 6, 2024	(TI=(psoriasis) OR AB=(psoriasis)) AND (TI=(depression) OR AB=(depression) OR TI=("depressive disorders") OR AB=("depressive disorders") OR TI=("mood depressive disorder") OR AB=("mood depressive disorder") OR TI=("Symptom, major depressive disorder") OR AB=("Symptom, major depressive disorder") OR TI=("Systematic Review") OR AB=("Systematic Review") OR TI=("Systematic Review and meta-analysis") OR AB=("Systematic Review and meta-analysis") OR TI=("Clinical trial") OR AB=("Clinical trial") OR TI=("Randomized Clinical Trial") OR AB=("Randomized Clinical Trial") OR TI=("Cross-section study") OR AB=("Cross-section study") OR TI=("Longitudinal study") OR AB=("Longitudinal study") OR TI=("Retrospective study") OR AB=("Retrospective study") OR TI=("Hospitalization") OR AB=("Hospitalization") OR TI=(psychotherapy) OR	N= 3,028

Bibliographic database	Search data	Search string	Results
		AB=(psychotherapy) OR TI=("SSRIs") OR AB=("SSRIs") OR TI=("tricyclic antidepressant") OR AB=("tricyclic antidepressant") OR TI=("Antidepressant therapy") OR AB=("Antidepressant therapy") OR TI=("cognitive behavioral therapy") OR AB=("cognitive behavioral therapy") OR TI=("non-pharmacological strategies") OR AB=("non-pharmacological strategies") OR TI=(anxiety) OR AB=(anxiety) OR TI=("anxiety disorders") OR AB=("anxiety disorders") AND PY=(1974-2023) AND (DT=("Article") OR DT=("Review")	
Pubmed	June 6, 2024	(psoriasis[Title/Abstract]) AND (depression[Title/Abstract] OR "depressive disorders"[Title/Abstract] OR "mood depressive disorder"[Title/Abstract] OR "Symptom, major depressive disorder"[Title/Abstract] OR "Systematic Review"[Title/Abstract] OR "Systematic Review and meta-analysis"[Title/Abstract] OR "Clinical trial"[Title/Abstract] OR "Randomized Clinical Trial"[Title/Abstract] OR "Cross-section study"[Title/Abstract] OR "Longitudinal study"[Title/Abstract] OR "Retrospective study"[Title/Abstract] OR Hospitalization[Title/Abstract] OR psychotherapy[Title/Abstract] OR SSRIs[Title/Abstract] OR "tricyclic antidepressant"[Title/Abstract] OR "Antidepressant therapy"[Title/Abstract] OR "cognitive behavioral therapy"[Title/Abstract] OR "non-pharmacological strategies"[Title/Abstract] OR anxiety[Title/Abstract] OR "anxiety disorders"[Title/Abstract]) AND ("1974"[Date - Publication] : "2023"[Date - Publication]) AND (("journal article"[Publication Type]) OR ("review"[Publication Type]))	N = 3574
